# Molar Pregnancy: Early Diagnosis, Clinical Management, and the Role of Referral Centers

**DOI:** 10.3390/diagnostics15151953

**Published:** 2025-08-04

**Authors:** Antônio Braga, Lohayne Coutinho, Marcela Chagas, Juliana Pereira Soares, Gustavo Yano Callado, Raphael Alevato, Consuelo Lozoya, Sue Yazaki Sun, Edward Araujo Júnior, Jorge Rezende-Filho

**Affiliations:** 1 Department of Gynecology and Obstetrics, School of Medicine, Federal University of Rio de Janeiro (UFRJ), Rua das Laranjeiras, No. 180, Laranjeiras, Rio de Janeiro 22240-003, RJ, Brazil; rezendef@me.ufrj.br; 2 Department of Maternal and Child Health, Postgraduate Program in Medical Sciences, School of Medicine, Fluminense Federal University (UFF), Niterói 24070-090, RJ, Brazil; rfalevato@gmail.com; 3 Postgraduate Program in Applied Health Sciences, University of Vassouras, Vassouras 27700-000, RJ, Brazil; lohaynemarinsteixeira@gmail.com (L.C.); marcelavchagas@gmail.com (M.C.); ju.psoares@hotmail.com (J.P.S.); 4 Discipline of Woman Health, Albert Einstein Israelite College of Health Sciences (FICSAE), Albert Einstein Israelite Hospital, São Paulo 05653-000, SP, Brazil; gycallado@gmail.com (G.Y.C.); araujojred@terra.com.br (E.A.J.); 5 Department of Pathology, Fluminense Federal University (UFF), Niterói 24070-090, RJ, Brazil; consuelolozoya@id.uff.br; 6 Department of Obstetrics, Paulista School of Medicine, Federal University of São Paulo (EPM-UNIFESP), Sao Paulo 04023-062, SP, Brazil; sueysun@gmail.com; 7 Discipline of Woman Health, Municipal University of São Caetano do Sul (USCS), São Caetano do Sul 09521-160, SP, Brazil

**Keywords:** molar pregnancy, clinical presentation, diagnosis, treatment, follow-up

## Abstract

Molar pregnancy (MP) is a gestational disorder resulting from abnormal fertilization, leading to atypical trophoblastic proliferation and the formation of a complete or partial hydatidiform mole. This condition represents the most common form of gestational trophoblastic disease (GTD) and carries a significant risk of progression to gestational trophoblastic neoplasia (GTN). Although rare in high-income countries, MP remains up to ten times more prevalent in low-income and developing countries, contributing to preventable maternal morbidity and mortality. This narrative review provides an updated, practical overview of the clinical presentation, diagnosis, treatment, and follow-up of MP. A key focus is the challenge of early diagnosis, particularly given the increasing frequency of first-trimester detection, where classical histopathological criteria may be subtle, leading to diagnostic errors. The review innovates by integrating advanced diagnostic methods—combining histopathology, immunohistochemistry using p57Kip2, Ki-67, and p53 markers, along with cytogenetic analysis—to improve diagnostic accuracy in early gestation. The central role of referral centers is also emphasized, not only in facilitating timely treatment and access to chemotherapy, but also in implementing standardized post-molar follow-up protocols that reduce progression to GTN and maternal mortality. By focusing on both advanced diagnostic strategies and the organization of care through referral centers, this review offers a comprehensive, practice-oriented perspective to optimize patient outcomes in GTD and address persistent care gaps in high-burden regions.

## 1. Introduction

Molar pregnancy (MP) is a gestational anomaly resulting from abnormal fertilization, leading to aberrant trophoblastic proliferation [1]. It represents the most common form of gestational trophoblastic disease (GTD) and is the primary risk factor for the development of gestational trophoblastic neoplasia (GTN)—a malignancy of placental origin [2]. Although considered rare in high-income countries, MP is 5 to 10 times more frequent in Brazil than in the United States and Europe, respectively, affecting approximately 1 in every 200–400 pregnancies in the Brazilian population [3,4].

**Figure 1:**
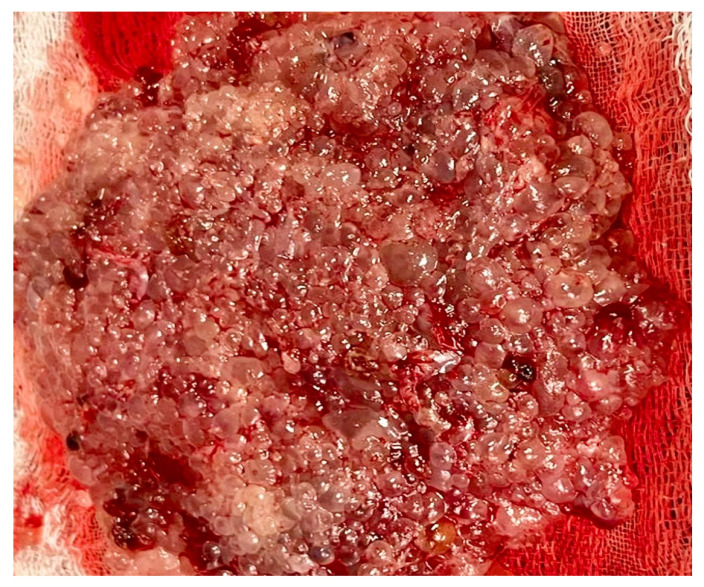
Macroscopic appearance of complete hydatidiform mole. Only the amorphous placental mass is visible, interspersed with translucent hydropic vesicles. Uterine evacuation was performed during the 8th week of gestation; thus, the vesicles are small, consistent with an early gestational-age mole. Note the absence of an embryo and embryonic appendages (such as yolk sac and umbilical cord).

**Figure 2:**
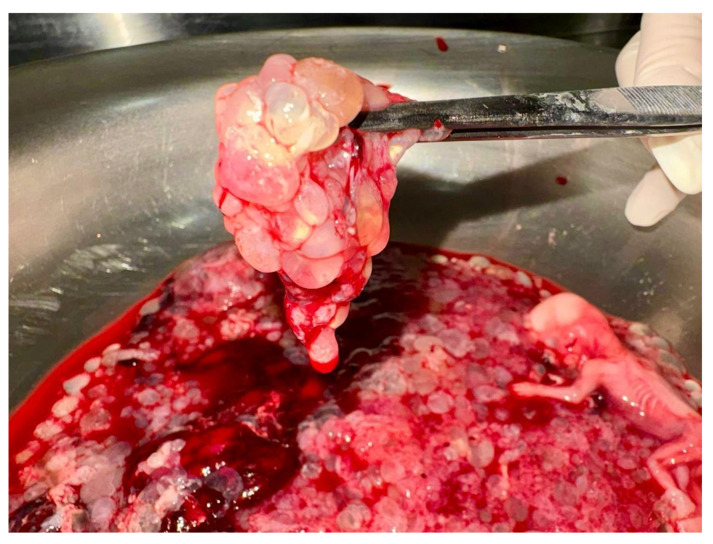
Macroscopic appearance of partial hydatidiform mole. This molar pregnancy was interrupted at 14 weeks’ gestation. The placenta shows numerous hydropic vesicles scattered over the maternal surface.

From etiopathogenic, clinical, and prognostic perspectives, MP manifests as two distinct syndromes: CHM and PHM. CHM results from the fertilization of an enucleate oocyte by a haploid sperm that subsequently duplicates its DNA or, less commonly, by two haploid sperm. In both scenarios, the resulting conception is a diandric diploid of parthenogenetic origin, leading to diffuse trophoblastic proliferation, marked hydropic edema of the chorionic villi, and absence of an embryo/fetus or its appendages Figure 1. In contrast, PHM typically arises from the fertilization of a genetically normal oocyte by two haploid sperm, or by a single sperm that duplicates its DNA, resulting in a diandric triploid conceptus with both maternal and paternal genetic contributions. This chromosomal composition allows for some embryonic and extraembryonic development. In rare cases where fetal development progresses, it is usually associated with multiple anomalies incompatible with life, and pregnancy rarely extends beyond the second trimester Figure 2. In such cases, the trophoblastic tissue displays less intense, focal proliferation, with minimally hydropic villi confined to trophoblastic islands [5].

Two hallmark features characterize MP. The first is the production of the biological tumor marker human chorionic gonadotropin (hCG), which plays a central role not only in diagnosis but also in follow-up, enabling assessment of malignant progression, monitoring of treatment response, and confirmation of remission. hCG levels are markedly elevated in CHM, while in PHM they may overlap with those observed in normal pregnancies. The second key feature is the preneoplastic potential of MP and its risk of progression to GTN—a risk estimated to be 4 to 20 times higher in CHM than in PHM [6].

With the widespread availability of ultrasonography in antenatal care, MP is increasingly being diagnosed at earlier gestational ages [7]. As a result, approximately half of the cases are now identified in asymptomatic patients. However, when diagnosis occurs near the end of the first trimester or later, clinical manifestations are more evident and may lead to obstetric near miss or even maternal death if not promptly managed [8].

Once MP is diagnosed, patients must be referred without delay to specialized referral centers [9,10]. In these centers, uterine evacuation—preferably by vacuum aspiration—is performed. This procedure alone achieves remission in approximately 80% of cases by removing the trophoblastic tissue and halting its proliferation. Nevertheless, even after successful evacuation, about 20% of patients will develop postmolar GTN and require further treatment to achieve remission [11].

Therefore, all patients diagnosed with MP must undergo structured postmolar follow-up, even after complete uterine evacuation, to enable early detection of GTN. This surveillance consists primarily of weekly hCG measurements to monitor for spontaneous remission or, conversely, detect persistent or malignant disease requiring intervention [1,2].

Early diagnosis, timely treatment, and systematic follow-up are essential to minimizing morbidity and preventing maternal deaths related to this condition. Although relatively infrequent, such outcomes are largely preventable and must be addressed—especially in low- and middle-income countries like Brazil, where structural barriers often delay access to specialized care [12].

Accordingly, the objective of this article is to detail strategies for the diagnosis of MP, recognition and management of medical complications, and to describe treatment and follow-up protocols aimed at the early detection of postmolar GTN. Emphasis is placed on the importance of specialized care provided at referral centers. This review aims to assist general practitioners and obstetricians/gynecologists—particularly those less familiar with this rare disease—by offering sound clinical and diagnostic guidance for managing this potentially life-threatening obstetric emergency during the first half of pregnancy.

## 2. Diagnosis of Molar Pregnancy

Early diagnosis is the primary strategy for preventing clinical complications of MP. Although approximately 50% of cases are now identified in asymptomatic patients during routine prenatal ultrasonography, vaginal bleeding remains the most frequently reported symptom and the leading cause for emergency imaging [7,12]. While early detection enables intervention before complications arise, it does not affect the oncological prognosis of MP, which is determined by intrinsic biological and genetic characteristics of the abnormal trophoblastic tissue [7].

Pregnancy should always be considered in the differential diagnosis of any woman of reproductive age with an active sexual life who presents with vaginal bleeding. In such cases, a simple qualitative hCG test—either serum or urinary—provides rapid and valuable diagnostic information. If pregnancy is confirmed, ultrasonography becomes a key tool to assess the etiology of first-trimester bleeding and to differentiate between types of miscarriage, ectopic pregnancy, or MP.

**Figure 3:**
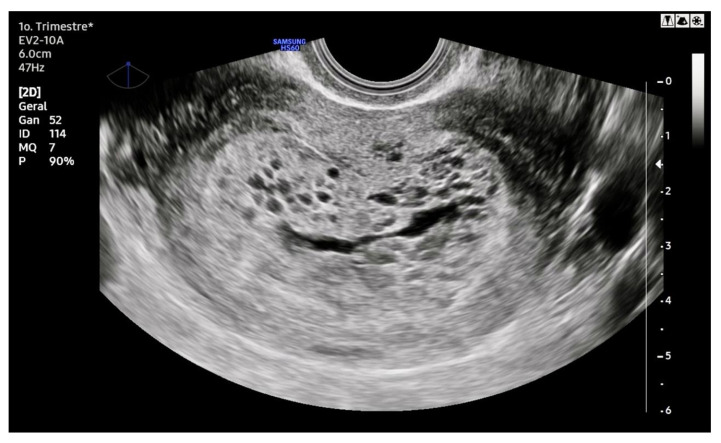
Ultrasound of complete hydatidiform mole. The endometrial cavity is filled with an anechoic mass exhibiting multiple cystic areas (representing hydropic vesicles). No embryonic or appendageal structures are seen, but the uterus appears enlarged for gestational age, measuring 12 cm at its greatest diameter in what corresponds to an 8-week pregnancy.

**Figure 4:**
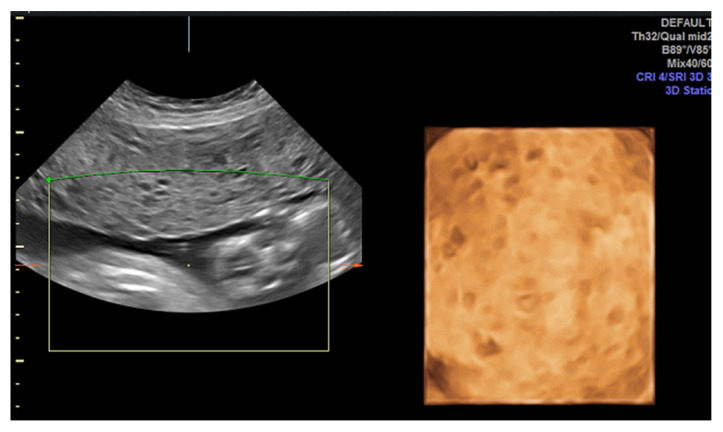
Ultrasound of partial hydatidiform mole. A nonviable fetus at 14 weeks’ gestation is visible, along with a placental region containing two large anechoic areas.

The classic ultrasonographic appearance of molar pregnancy (MP) is a heterogeneous intrauterine mass containing numerous anechoic cystic spaces, often described as having a “snowstorm” or “cluster of grapes” pattern Figure 3, which corresponds to hydropic and edematous chorionic villi [13]. In cases of CHM, this pattern typically occurs in the absence of a gestational sac or embryo/fetus, and the uterus is usually enlarged for the gestational age. Bilateral theca-lutein cysts may also be observed, secondary to ovarian hyperstimulation caused by elevated hCG levels. This occurs because the hCG molecule shares an alpha subunit homologous to that of follicle-stimulating hormone (FSH) and luteinizing hormone (LH), thereby stimulating follicular development [14]. In contrast, the ultrasonographic findings in PHM tend to be subtler. A gestational sac is usually present and may contain an embryo or fetus with structural anomalies and intrauterine growth restriction Figure 4. The placenta typically appears thickened and displays focal cystic spaces, reflecting milder hydropic changes than those seen in CHM [15].

However, these classical findings are more commonly observed toward the end of the first trimester or the beginning of the second trimester. At earlier gestational ages, sonographic diagnosis of both CHM and PHM can be particularly challenging. In early CHM, the characteristic multicystic pattern may not yet be evident. Instead, the uterus may appear to contain a homogeneous echogenic mass without a distinct gestational sac or embryo. Occasionally, a small anechoic area may mimic a gestational sac but lacks a yolk sac or embryonic structures. These features can resemble an anembryonic pregnancy or early pregnancy loss. In early PHM, ultrasonographic findings may resemble those of a missed abortion: a gestational sac is typically present, often containing an embryo without cardiac activity. The placenta may appear slightly enlarged with subtle cystic spaces, which are frequently too inconspicuous to raise immediate suspicion for MP. As a result, PHM is often underdiagnosed and only recognized when uterine contents are submitted for histopathological evaluation [14].

Histopathological diagnosis remains a critical and sometimes challenging step in identifying MP, particularly in early gestational age specimens where classical morphological features may be subtle. In CHM, typical histopathological findings include diffuse villous edema, circumferential trophoblastic proliferation, absence of embryonic or fetal tissues, and central cistern formation within the chorionic villi. However, in early gestation CHM, these features may be less conspicuous, leading to diagnostic uncertainty. PHM is characterized by a mixture of normal and abnormal villi, with focal villous edema, irregular scalloped borders, trophoblastic pseudoinclusions, and the presence of embryonic or fetal remnants. In early PHM, these changes can be minimal, increasing the overlap with non-molar hydropic abortions.

**Figure 5:**
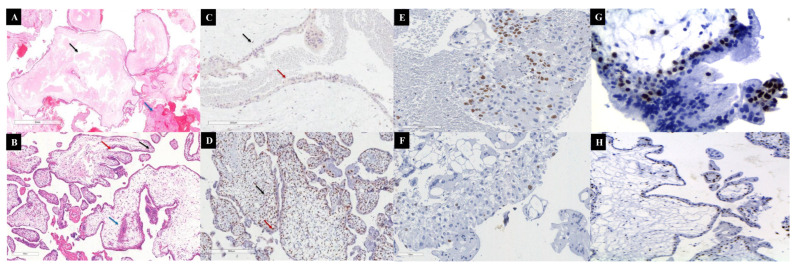
Pathological findings of hydatidiform moles. (**A**) Representative case of complete hydatidiform mole (CHM). Note the presence of enlarged, hydropic chorionic villi with central cistern formation (black arrow), avascular stromal cores, and moderate circumferential trophoblastic hyperplasia (blue arrow) (Stain: Hematoxylin–Eosin). (**B**) Representative case of partial hydatidiform mole (PHM), showing a dual villous population composed of enlarged hydropic villi interspersed with smaller, more fibrotic ones. Finger-like projections (black arrow), trophoblastic invaginations (blue arrow), and intravillous inclusions (red arrow) are also observed (Stain: Hematoxylin–Eosin). (**C**) Absence of p57Kip2 immunostaining in both the villous cytotrophoblast (red arrow) and stromal cells (black arrow), consistent with the androgenetic origin and diagnosis of CHM. (**D**) Positive nuclear expression of p57Kip2 in cytotrophoblasts (red arrow) and villous stromal cells (black arrow), supporting the diagnosis of PHM. (**E**) Immunohistochemical staining for Ki-67 in CHM reveals a high proliferative index, with strong and widespread nuclear positivity in cytotrophoblastic and syncytiotrophoblastic cells, indicating intense mitotic activity. (**F**) In PHM, Ki-67 staining demonstrates a moderate proliferative index, with predominantly focal nuclear positivity in cytotrophoblasts and markedly lower labeling compared to CHM. (**G**) p53 immunostaining in CHM shows increased nuclear expression, particularly in cytotrophoblasts (semiquantitative score: 3+), with a diffuse to moderate distribution, reflecting deregulated cell cycle control and reduced apoptotic susceptibility. (**H**) In PHM, p53 expression is weak and focal (semiquantitative score: 1+), mainly restricted to cytotrophoblastic cells, with minimal to absent staining in syncytiotrophoblasts, consistent with the lower proliferative potential of PHM.

To overcome these diagnostic challenges, immunohistochemistry (IHC) has become an essential adjunct. The p57Kip2 marker, a maternally expressed gene product, shows strong nuclear positivity in cytotrophoblasts and villous stromal cells in PHM and non-molar pregnancies, while it is absent in CHM due to its androgenetic origin. The Ki-67 labeling index is typically higher in CHM compared to PHM and non-molar gestations, reflecting increased trophoblastic proliferation. p53 expression patterns may also assist, with overexpression more commonly observed in CHM, as can be seen in Figure 5.

Cytogenetic analysis further refines the diagnosis: CHM usually exhibits diploid androgenetic karyotypes (46,XX, or less frequently 46,XY), while PHM typically presents as diandric triploidy (69,XXY or 69,XXX). Techniques such as short tandem repeat (STR) genotyping provide definitive genetic confirmation by distinguishing androgenetic from biparental contributions.

Despite these tools, challenges persist, particularly in resource-limited settings where access to IHC and molecular diagnostics may be restricted. Future developments aim to address these limitations. One promising area is the application of circulating cell-free DNA analysis, which may enable non-invasive detection of molar pregnancies through assessment of abnormal genomic imprinting patterns or ploidy status. Additionally, advanced digital pathology combined with artificial intelligence-based image analysis is being explored to enhance pattern recognition in histopathological slides, potentially reducing observer variability.

In conclusion, although histopathology supported by IHC and cytogenetic analysis currently represents the gold standard for MP diagnosis, ongoing research into minimally invasive and automated technologies holds promise for improving diagnostic accuracy and accessibility in the near future. Table 1 below provides a comprehensive comparison between CHM and PHM, highlighting their clinical, histopathological, molecular, and genetic characteristics.

## 3. Diagnosis and Treatment of Clinical Complications of Molar Pregnancy

Once molar pregnancy (MP) is diagnosed—particularly through ultrasonography—the patient should be promptly referred for treatment. Uterine evacuation halts trophoblastic proliferation, which is responsible for the typical clinical complications associated with delayed diagnosis and is more commonly observed in pregnancies diagnosed after the first trimester [1,2].

When MP is diagnosed beyond the first trimester, it may be associated with a range of potentially severe complications. These result from abnormal trophoblastic proliferation, extremely elevated levels of hCG, and local invasion or systemic dissemination of trophoblastic tissue. Below, we outline the main clinical complications of MP and their respective management strategies [8,12].

### 3.1. Uterine Hemorrhage

**Figure 6:**
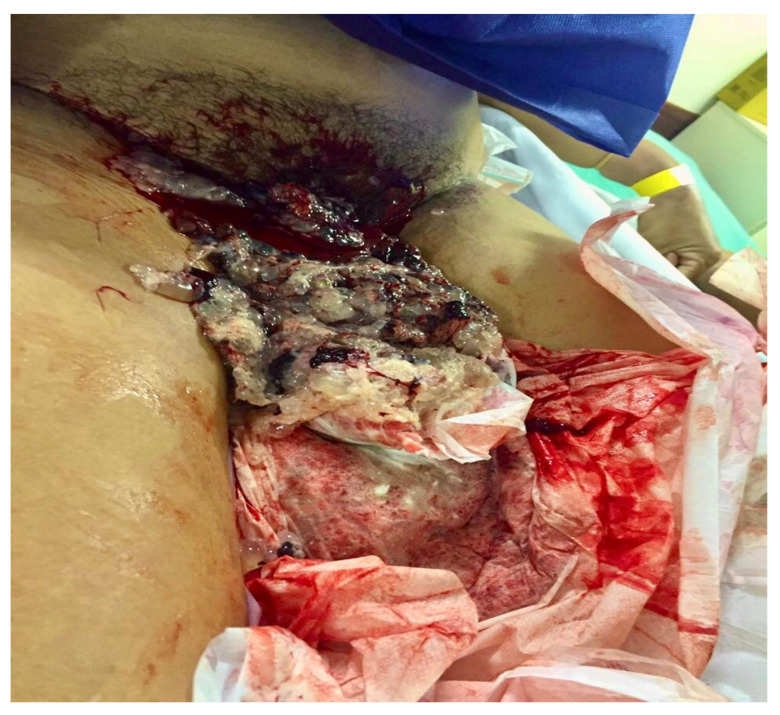
Image of a patient with 17 weeks of amenorrhea and a uterine fundal height of 22 cm, referred to the Gestational Trophoblastic Disease Referral Center at the Maternity School of Federal University of Rio de Janeiro with a diagnosis of molar pregnancy. Upon admission, the patient presented with profuse vaginal bleeding and expulsion of vesicles—a pathognomonic sign of the disease. Note the significant diagnostic delay, which led to clinical complications and the need for multiple blood transfusions, characterizing an obstetric near miss.

Hemorrhage is the most frequent complication of MP and may range from mild to severe, occasionally accompanied by vesicular expulsion and risk of hypovolemic shock Figure 6. The primary cause is the presence of trophoblastic material within the endometrial cavity. In such cases, primary or repeat uterine evacuation is indicated. Ultrasonographic or hysteroscopic guidance can improve surgical precision and reduce procedural risks [16].

Management includes immediate hemodynamic stabilization, close monitoring of vital signs, large-bore intravenous access, volume resuscitation with crystalloids, and blood transfusion if necessary. Temporary hemostatic measures, such as uterine tamponade, may also be employed. While the Bakri balloon is generally too large for the early gestational uterus, modified Foley catheters may be effective and allow time for stabilization or transfer to a specialized center.

Emergency uterine evacuation should not be delayed. If the patient is hemodynamically unstable or if uterine perforation is suspected, exploratory laparotomy should be considered to assess the uterus and pelvic organs. Oxytocin may be administered after the initiation of uterine evacuation but should be avoided beforehand due to the risk of trophoblastic embolization [17].

In more severe cases, as a last conservative measure to preserve the uterus, vascular ligation (of the uterine or internal iliac arteries) may be performed. This technique rapidly reduces uterine blood flow and achieves hemostasis. Minimally invasive uterine artery embolization (UAE), performed by interventional radiology, is another option for persistent bleeding. UAE achieves immediate bleeding control in over 90% of cases, with shorter recovery times, minimal blood loss, and a reduced need for major surgery (e.g., hysterectomy) [18].

**Figure 7:**
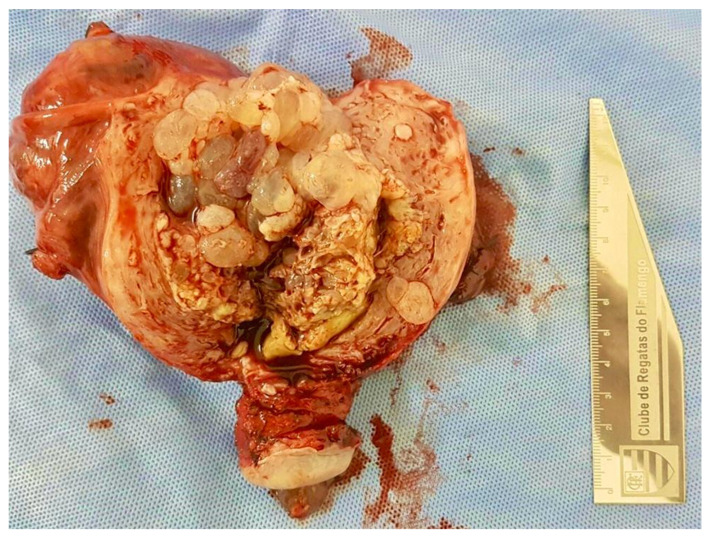
Image of a molar uterus. Hysterectomy was performed in a patient diagnosed with molar pregnancy at 48 years of age. Given advanced maternal age and completed parity, hysterectomy was chosen to reduce the risk of postmolar trophoblastic neoplasia. However, during follow-up, hCG levels remained elevated, and chemotherapy was required to achieve remission. This case highlights the necessity of post-hysterectomy hormonal surveillance.

In selected patients, total abdominal hysterectomy (TAH) may be indicated in cases of life-threatening hemorrhage, particularly in women of advanced maternal age or with completed childbearing Figure 7. However, even after hysterectomy, there remains a small but consistent risk of postmolar gestational trophoblastic neoplasia (GTN). Therefore, patients who undergo TAH must continue hormonal surveillance as part of postmolar follow-up [19].

### 3.2. Early-Onset Preeclampsia (Before 20 Weeks’ Gestation)

Preeclampsia diagnosed before 20 weeks of gestation is highly suggestive of MP. It should be considered in the presence of hypertension (systolic blood pressure ≥ 140 mmHg and/or diastolic ≥ 90 mmHg on at least two occasions, four hours apart, in a previously normotensive woman) accompanied by at least one of the following: proteinuria (≥300 mg of protein in a 24-h urine sample, or protein/creatinine ratio ≥ 0.3 in a spot urine sample, or ≥1+ on a urine dipstick); maternal organ dysfunction—such as renal impairment (serum creatinine > 1.1 mg/dL or doubling of baseline in the absence of other renal disease), hepatic involvement (transaminases > 2× upper limit of normal, with or without epigastric or right upper quadrant pain), thrombocytopenia (platelet count < 100,000/mm^3^), pulmonary edema, or neurological symptoms (persistent headache, visual disturbances such as scotomas or blurred vision, hyperreflexia, or seizures) [20].

This is a severe clinical condition requiring hospitalization and immediate treatment. Blood pressure should be controlled using antihypertensives such as hydralazine or nifedipine. In the presence of severe features—such as persistent headache, scotomas, epigastric pain, systolic pressure > 160 mmHg, diastolic > 110 mmHg, or laboratory signs of severe disease—magnesium sulfate is indicated for seizure prophylaxis [20].

The only definitive treatment in such cases is uterine evacuation, performed once the patient is clinically stabilized. After removal of the trophoblastic tissue, the signs and symptoms of preeclampsia typically resolve within a few days [16].

### 3.3. Hyperthyroidism

Due to the agonist effect of hCG on thyroid-stimulating hormone (TSH) receptors, transient hyperthyroidism may occur in MP as a result of markedly elevated hCG levels from trophoblastic hyperplasia [21].

Mild cases are common and self-limited; however, severe presentations—including thyroid storm—may occur. In mild to moderate cases, observation and close monitoring are generally sufficient. In severe cases—characterized by fever (>38.5 °C), tachycardia (>140 bpm), hypertension, agitation, confusion, delirium or coma, nausea/vomiting, diarrhea, and potential progression to cardiac or respiratory failure—hospitalization is mandatory [21].

In intensive care, patients must be monitored for risk of thyrotoxic crisis. Supportive management includes temperature control (via physical methods and antipyretics), cautious intravenous hydration (to avoid pulmonary edema due to hyperdynamic circulation), oxygen therapy, and ventilatory support if needed. Specific pharmacologic treatment includes:

- Beta-blockade: propranolol 40–80 mg orally every 6 h or 1–2 mg IV every 10 min until heart rate is controlled;
- Inhibition of hormone synthesis: propylthiouracil 500–1000 mg orally as a loading dose, then 250 mg every 6 h;
- Inhibition of hormone release: potassium iodide, 5 drops orally every 6 h, administered 1 h after propylthiouracil;
- Reduction of peripheral T4-to-T3 conversion: hydrocortisone 100 mg IV every 8 h.

Refractory cases may require plasmapheresis [21]. Clinical improvement and remission of thyroid dysfunction occur only after uterine evacuation; therefore, once the patient is clinically stabilized, prompt uterine aspiration should be performed [16].

### 3.4. Acute Respiratory Distress Syndrome/Trophoblastic Embolization

Acute respiratory distress syndrome (ARDS) is a rare but life-threatening complication of MP, particularly following uterine evacuation. It is most commonly caused by trophoblastic embolization to the lungs but may also result from severe hyperthyroidism, hyperdynamic circulation, or iatrogenic fluid overload. Judicious use of uterotonics—especially misoprostol—can reduce the risk of trophoblastic embolization during evacuation [22].

When fragments of trophoblastic tissue enter the maternal circulation and reach the pulmonary capillaries, they may cause both mechanical obstruction and, more importantly, endothelial injury with the release of proinflammatory cytokines. These mediators increase capillary permeability, leading to alveolar edema, hypoxemia, and alveolar collapse—triggering the systemic inflammatory cascade that defines MP-associated ARDS [23].

This condition has an abrupt onset, typically developing within minutes to hours after uterine evacuation. Clinically, patients present with sudden, severe dyspnea, tachypnea, tachycardia, refractory hypoxemia, cyanosis, and diffuse inspiratory crackles. Without rapid intervention, the condition can progress to pulmonary edema, hypotension, shock, and cardiorespiratory arrest [24].

Although the diagnosis of ARDS in this context is primarily clinical, ancillary tests are helpful. Arterial blood gas analysis reveals severe hypoxemia (PaO_2_ < 60 mmHg on FiO_2_ > 0.5; PaO_2_/FiO_2_ ratio ≤ 300 mmHg), early respiratory alkalosis, and metabolic acidosis in advanced stages. Chest radiography or computed tomography typically shows bilateral diffuse pulmonary infiltrates without cardiomegaly, consistent with non-cardiogenic pulmonary edema.

Management requires intensive care, including ventilatory support (high-flow oxygen or mechanical ventilation with positive end-expiratory pressure to maintain O_2_ saturation > 90%), fluid management (with strict monitoring to avoid volume overload), and vasopressor support when hypotension occurs. The use of corticosteroids remains controversial, though many clinicians administer methylprednisolone to reduce pulmonary inflammation [22,23].

With early recognition and appropriate management, prognosis is generally favorable; however, maternal mortality remains high in untreated or rapidly progressive cases [16].

## 4. Primary Treatment of Molar Pregnancy

Uterine evacuation via vacuum aspiration is currently regarded as the standard treatment for molar pregnancy (MP) [1,2]. This surgical procedure is effective, safe, and associated with a lower risk of complications compared to conventional sharp curettage (which carries a higher risk of incomplete evacuation and uterine perforation) or the use of misoprostol (which is linked to incomplete evacuation, increased risk of trophoblastic embolization, and a greater need for chemotherapy during postmolar follow-up) [17].

**Figure 8:**
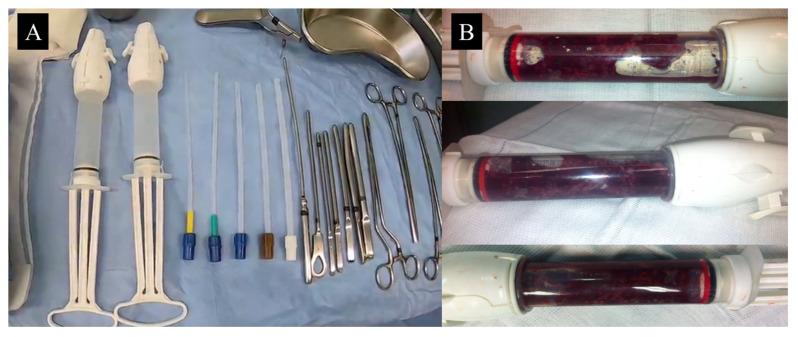
(**A**) Manual intrauterine aspiration syringes and various sizes of aspiration cannulas. This is the most widely used equipment in Brazil for vacuum aspiration in definitive treatment of molar pregnancy. (**B**) Syringe filled with molar material freshly aspirated from the uterine cavity.

Uterine evacuation can be performed using either electric or manual vacuum aspiration Figure 8A,B. Both methods are equally effective for the removal of molar tissue, although manual aspiration is more widely available in Brazil [25].

**Figure 9:**
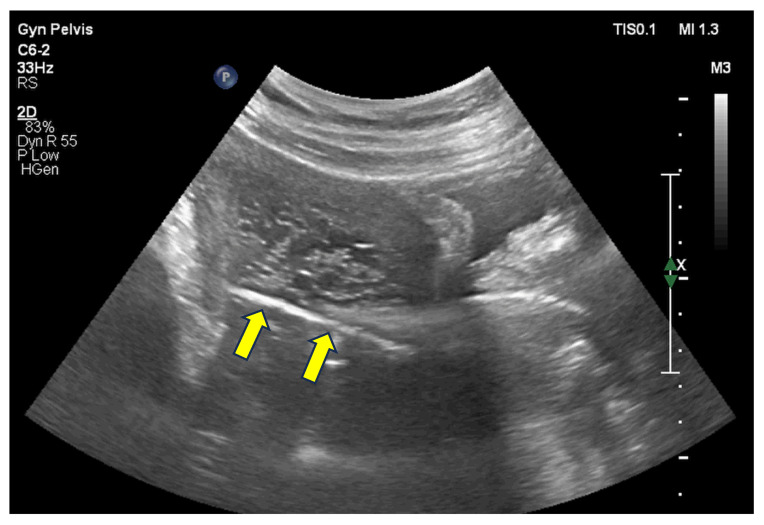
Pelvic ultrasound used to guide uterine evacuation for the treatment of molar pregnancy. Yellow arrows indicate the uterine aspiration cannula suctioning molar tissue. Ultrasound guidance during evacuation reduces the risk of uterine perforation and minimizes residual trophoblastic tissue.

**Figure 10:**
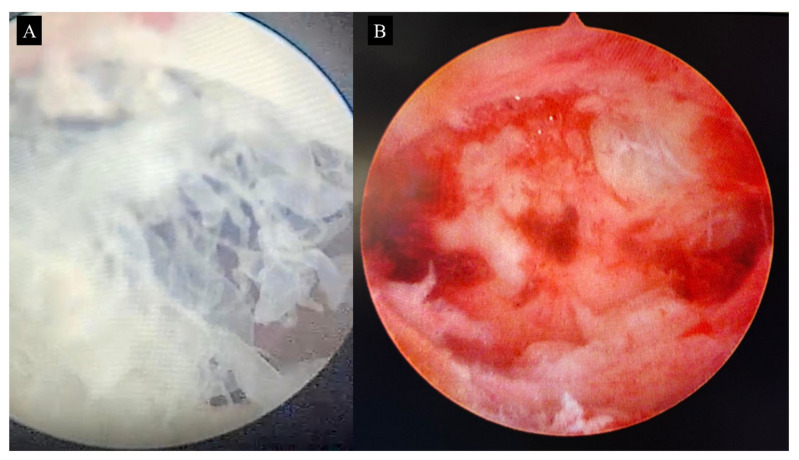
Surgical hysteroscopy showing hydropic vesicles within the endometrial cavity (**A**). Following routine uterine evacuation, a repeat evaluation confirms complete removal of all trophoblastic tissue, ensuring the success of the procedure (**B**).

Surgery performed by an experienced team ensures careful cervical dilation, minimizing the risk of uterine perforation and optimizing complete evacuation. The cannula size is selected according to estimated uterine volume, typically ranging from 7 to 9 mm. Gentle aspiration should be performed using circular movements of the cannula, ideally under pelvic ultrasound guidance Figure 9 [25,26]. Additionally, surgical hysteroscopy can help identify the site of molar implantation and detect any residual tissue which, depending on the quantity, may require repeat aspiration or delicate removal with hysteroscopic forceps Figure 10A,B [27].

Revision curettage should only be performed by skilled operators and only when retained molar tissue is suspected. In most cases, it should be avoided due to the risk of uterine perforation.

In uncomplicated procedures, hospital discharge can occur 6 to 12 h after recovery from anesthesia. Home prescriptions should include standard analgesia and hormonal contraception, when not contraindicated. Contraceptive use must be maintained throughout the postmolar follow-up to preserve the accuracy of tumor marker interpretation and ensure early diagnosis of postmolar gestational trophoblastic neoplasia (GTN) [28,29].

## 5. Postmolar Follow-Up

Postmolar follow-up is an essential step in MP management, enabling early identification of patients who will undergo spontaneous remission and those at risk of persistent disease or progression to GTN [1,2].

Follow-up is primarily based on weekly serum hCG measurements. To avoid technical variability, the same laboratory assay should be used throughout the surveillance period [30].

Remission is defined after three consecutive weekly hCG values below 5 IU/L. In cases of PHM, a repeat hCG test should be performed 30 days after remission. If the result remains normal, the patient may be discharged. For CHM, hCG levels should be monitored for 3 months if remission occurs within 56 days of uterine evacuation, or for 6 months if it occurs afterward [1,2].

Once follow-up is completed, patients may attempt a new pregnancy. Most women with a history of MP go on to have normal, healthy pregnancies with low risk of complications. Women who conceive after completing follow-up with normalized hCG levels and no signs of GTN have an excellent reproductive prognosis. The recurrence risk of MP is low (approximately 1–2%) but remains higher than in the general population. After two consecutive molar pregnancies, the recurrence risk increases to 10–20%. Future pregnancies should be planned, and early ultrasound should confirm fetal viability and placental morphology [31].

Genetic testing plays a critical role in evaluating recurrent MP, helping to identify hereditary predispositions that increase recurrence risk. Familial hydatidiform mole, a rare inherited form of gestational trophoblastic disease, is associated with mutations in specific genes, particularly NLRP7, KHDC3L, and, to a lesser extent, PADI6. The NLRP7 gene is most frequently implicated, accounting for approximately 75% of familial cases, and plays roles in inflammasome function and maternal epigenetic regulation—both essential for normal placental development. Identification of these mutations not only confirms the diagnosis but also guides genetic counseling and personalized clinical management, enabling close monitoring and early intervention to improve reproductive outcomes [32,33].

## 6. Diagnosis of Postmolar Gestational Trophoblastic Neoplasia

The diagnosis of postmolar GTN is based on clinical, laboratory, and occasionally histopathological criteria, with serial hCG monitoring serving as the primary diagnostic tool. GTN is the only known human malignancy in which diagnosis is not primarily histopathological but biochemical [1,2].

According to the International Federation of Gynecology and Obstetrics (FIGO) and the World Health Organization (WHO), postmolar GTN is diagnosed when at least one of the following criteria is met [34]:

- Plateaued hCG levels (variation < 10%) across four consecutive weekly measurements over 3 weeks;
- Rising hCG values (increase of ≥10%) across three consecutive weekly measurements over at least 2 weeks;
- Histopathological diagnosis of choriocarcinoma, placental site trophoblastic tumor, or epithelioid trophoblastic tumor.

In such cases, the treatment of choice is chemotherapy, which achieves excellent cure rates—even in chemoresistant and metastatic disease [1,2].

## 7. Importance of Referral Centers for Gestational Trophoblastic Disease

Referral centers play a fundamental role in managing patients with MP and its complications, as well as in the early diagnosis, treatment, and follow-up of GTN. Centralizing care in these specialized services improves outcomes, reduces complications, and is considered a cost-effective public health strategy [35].

These centers are staffed by professionals trained to identify early clinical, histopathological, and imaging findings of GTD. They offer access to appropriate diagnostic resources such as hCG testing, histopathology, ultrasound, and advanced imaging (CT and MRI) for cancer staging [9,10].

In addition, the presence of multidisciplinary teams—including specialized nurses, social workers, and psychologists—ensures emotional support for women who may experience grief over pregnancy loss or fear of cancer and death. These professionals help reinforce adherence to follow-up, address logistical barriers, and promote consistent contraceptive use, thereby minimizing treatment abandonment [36,37,38].

Studies show that care in specialized centers is associated with shorter time to GTN diagnosis, quicker treatment initiation, reduced need for multi-agent chemotherapy, lower treatment toxicity, and higher cure rates (>98%)—even in metastatic cases. These findings underscore the role of referral centers as the ideal setting for managing GTD [9,10].

## 8. Conclusions

Although rare, molar pregnancy remains a potentially life-threatening condition when not diagnosed and managed promptly. Its preneoplastic nature and potential for progression into GTN demand heightened vigilance and early intervention, particularly in primary care and obstetric emergency settings. Effective management depends on early diagnosis, appropriate treatment, and rigorous follow-up. However, challenges persist—including delays in referral, regional disparities in access to specialized care, and the need to strengthen health infrastructure and training. Addressing these barriers is essential to fully realize the benefits of referral center-based care and ensure equitable outcomes for all women affected by MP. Future research should focus on optimizing postmolar follow-up protocols, evaluating the effectiveness of telemedicine and digital health tools in low-resource settings, and expanding the evidence base for early diagnostic strategies and personalized treatment approaches in GTD. In this context, referral centers are pivotal for ensuring timely access to uterine evacuation, standardized chemotherapy protocols, systematic hCG monitoring, and integrated psychosocial support—all critical to reducing complications and improving outcomes. These centers provide not only technical expertise but also compassionate, continuous care. Strengthening and expanding referral networks represents a strategic public health initiative to reduce maternal morbidity and mortality by ensuring that all women with GTD have access to specialized, evidence-based, and multidisciplinary management.

## Figures and Tables

**Table 1:** Comprehensive comparison between complete and partial hydatidiform moles: clinical, histopathological, molecular and genetic features.

Feature	Complete Hydatidiform Mole	Partial Hydatidiform Mole
Clinical presentation	Vaginal bleeding; uterus larger than gestational age; markedly elevated β-hCG; absent fetal parts	Vaginal bleeding; uterus appropriate or slightly enlarged; mildly elevated β-hCG; fetal parts may be present
Ultrasound findings	“Snowstorm” or “cluster of grapes” appearance; no embryo or fetus	Cystic, thickened placenta; abnormal fetus may be present; “Swiss cheese” appearance
Histopathology	Diffusely hydropic villi with central cisterns; circumferential and diffuse trophoblastic hyperplasia; absence of fetal tissue	Mixed villous population with scalloped hydropic villi; trophoblastic inclusions; focal, mild trophoblastic hyperplasia; fetal tissue often present
p57Kip2 immunostaining	Negative in cytotrophoblasts and villous stromal cells	Positive nuclear staining in cytotrophoblasts and stromal cells
Ki-67 immunostaining	High proliferation index; strong nuclear positivity in cytotrophoblasts and syncytiotrophoblasts	Moderate proliferation index; mainly in cytotrophoblasts; lower than CHM
p53 immunostaining	Strong and diffuse nuclear expression, mainly in cytotrophoblasts (3+)	Weak and focal nuclear staining, mainly in cytotrophoblasts (1+)
Cytogenetic profile	Diploid, androgenetic (46,XX or 46,XY; entirely paternal)	Triploid, diandric (69,XXY; 69,XXX; 69,XYY; two paternal, one maternal genome)
Risk of postmolar GTN	15–20%	<5%

## Data Availability

The data presented in this study are available on request from the corresponding author.
